# Interleukin-35 and Thymoquinone nanoparticle-based intervention for liver protection against paracetamol-induced liver injury in rats

**DOI:** 10.1016/j.sjbs.2023.103806

**Published:** 2023-09-06

**Authors:** Maisa Siddiq Abduh, Sultan Ayesh Mohammed Saghir, Naif Ahmed Al-Gabri, Ahmad Faheem Ahmeda, Mouaadh Abdelkarim, Saleh Mohammad Aldaqal, Mohammed Abdullah Alshawsh

**Affiliations:** a Immune Responses in Different Diseases Research Group, Department of Medical Laboratory Sciences, Faculty of Applied Medical Sciences, King Abdulaziz University, Jeddah 21589, Saudi Arabia; b Center of Excellence in Genomic Medicine Research, King Abdulaziz University, Jeddah 21589, Saudi Arabia; c Department of Medical Analysis, Princess Aisha Bint Al-Hussein College of Nursing and Medical Sciences, Al-Hussein Bin Talal University, Ma’an 71111, Jordan; d Laboratory of Salam Veterinary Group, Burydha 51911, Saudi Arabia; e Department of Pathology, Faculty of Veterinary Medicine, Thamar University, Thamar 124401, Yemen; f Department of Basic Medical Sciences, College of Medicine, Ajman University, Ajman 346, United Arab Emirates; g Center of Medical and Bio-allied Health Sciences Research, Ajman University, Ajman 346, United Arab Emirates; h College of General Education, University of Doha for Science and Technology, Jelaiah Street, Duhail North, 24449 Doha, Qatar; i Immune Responses in Different Diseases Research Group, Department of Surgery, Faculty of Medicine, King Abdulaziz University, Jeddah 21589, Saudi Arabia; j School of Clinical Sciences, Faculty of Medicine, Nursing and Health Sciences, Monash University, 246 Clayton Road, Clayton, VIC, 3168, Australia; k Department of Pharmacology, Faculty of Medicine, Universiti Malaya, 50603 Kuala Lumpur, Malaysia

**Keywords:** Interleukin-35, Thymoquinone, Liver injury, Hepatotoxicity, Paracetamol

## Abstract

Paracetamol (PAR) is a commonly used antipyretic and analgesic agent, but its excessive usage can induce liver damage and major health consequences. Interleukin-35 (IL-35) is utilized to treat immunological disorders, intestinal illness, arthritis, allergic disease, hepatitis, and cancer. Thymoquinone (THYO) is also effective against a wide range of disorders. Consequently, this study sought out to explore the ameliorative effects of IL-35 and THYO against PAR-induced hepatotoxicity in rats. Sixty male rats were separated into six groups (10 rats/group): I control (0.5 mL NaCl, 0.9%/rat via oral gavage); II (IL-35), and III (TYHO) received intraperitoneal (i.p) injection of IL-35 (200 ng/kg) or THYO (0.5 mg/kg), respectively. Group IV (PAR) received 600 mg/kg of PAR orally; V (PAR + IL-35) and VI (PAR + TYHO); rats received 600 mg/kg of PAR orally and i.p injection of IL-35 (200 ng/kg) or THYO (0.5 mg/kg), respectively. Administration of IL-35 or THYO markedly mitigated the increasing in the levels of liver parameters triggered by PAR and noticeable enhancement of antioxidant and immunological markers were observed. Additionally, IL-35 or THYO decreased TNF-α, NF-κB, IL-10, IL-6 and IFN-γ in contrast to the PAR control group. Moreover, levels of Capase-3, and cytochrome *C* were significantly reduced by THYO or IL35, while, levels of Bcl-2 were markedly increased. Furthermore, significant downregulation of IL1-β, TNF-α, TGF-β, and Caspas-3 genes, as well as significant upregulation of Bcl-2 and IL-10 expression were detected. In conclusion, IL-35 and THYO insulated liver from PAR toxicity by mitigating oxidative stress, tissue damage, inflammation, and apoptosis.

## Graphical abstract

**Figure:**
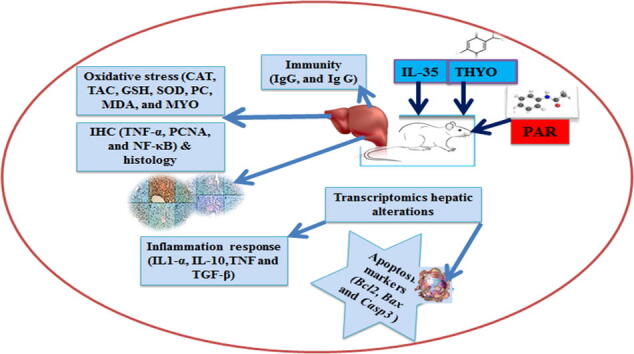

## 1 Introduction

Paracetamol (PAR) is a frequently preferred antipyretic and analgesic agent when used at therapeutic doses (Salman et al., 2020, Thrall et al., 2012). Its usage at higher doses could result in PAR toxicity due to the accumulation of toxic metabolites such as N-acetyl-p-benzoquinone (NAPQI). NAPQI is detoxified by intracellular glutathione in normal dose, but in overdoses, the GSH stores become depleted, leading to hepatotoxicity, liver damage, inflammation, and liver failure (Islam et al., 2021, Salman et al., 2020). Oxidative stress, steatosis, steatohepatitis, and liver dysfunction are the primary initiators of both acute and chronic PAR liver damage (Tejo, 2021, El-Boshy et al., 2019, Awad et al., 2018).

The immune cells produce a diverse array of biological products that allow them to interact and coordinate specialized immune responses to protect against diseases and infections (Omenesa Bello et al., 2018, Yoshimoto and Yoshimoto, 2013). One of the biological products is cytokines, which may act as significant mediators in dissemination of several infections, autoimmune syndromes, allergic illnesses, and neoplasms (Omenesa Bello et al., 2018). In these circumstances, various cytokines are released to regulate the host's cellular reactions to the invasive infections or irritating stimuli (Yoshimoto and Yoshimoto, 2013). Numerous investigations have shown that cytokines have s crucial effect in treating a wide range of ailments, including heart disease, autoimmune disorders, systemic lupus erythematosus, Hashimoto's thyroiditis, and arthritis (Lim et al., 2016, Egwuagu et al., 2015, Belladonna and Grohmann, 2013).

IL-35 is a heterodimeric cytokine of the IL-12 family that is used in disease model investigations as an immune-regulatory cytokine (Kopf et al., 2010). IL-35 boosts the immunity and defends against damage and inflammation of liver commenced due to oxidative stress (Egwuagu et al., 2015). It could exert its effect for protecting the liver by increasing the T-regulatory cells ratio, causing a surge in the anti-inflammatory cytokines and inhibition of immune cell proliferation (Hu et al., 2021). Additionally, IL-35 is primarily released by immune cells, including the recently discovered B regulatory cells (CD4 + CD25 + ). However, the recent researches have shown that the liver, bone marrow, thymus, and blood tissues all exhibit substantial levels of IL-35 expression (Li et al., 2020, Zhang et al., 2021). Earlier revisions illustrated that IL-35 has various roles in disease pathogenesis such as rheumatoid arthritis (Ning et al., 2015), autoimmune encephalomyelitis, uveitis (Wang et al., 2014), Hashimoto’s thyroiditis (Yilmaz et al., 2016), multiple low dose streptozotocin (Singh et al., 2015), allergic rhinitis (Suzuki et al., 2017), liver disease (Yang et al., 2020a), cardiovascular diseases (Posadas-Sánchez et al., 2017), and asthma (Kanai et al., 2017). Anti-inflammatory and pro-inflammatory processes are involved in the developing liver disorders, suggesting that IL-35 may produce a noteworthy effect in the advance of liver diseases via immunosuppressive regulations (Hu et al., 2021, Hoshino et al., 2020).

Furthermore, *in vivo* and *in vitro* and investigations revealed that IL-35 may suppress liver regeneration and hepatocyte proliferation (Liu et al., 2011). Some of these findings also point to IL-35 as a viable pharmaceutical target for reducing the advancement of liver ailments; however further study is still needed to understand how it works. Overall, researches display that IL-35 has a tremendous impact on immune modulation during the progress of chronic hepatitis, liver fibrosis, liver cirrhosis (LC), and hepatocellular carcinoma (HCC) development (Hu et al., 2021). Moreover, a previous in-depth study suggests that IL-35 can be utilized as a potential management approach to slow down the advancement of liver disorders, but more research is still needed to uncover its mechanism of action (Hu et al., 2021).

At the same time, due to the safety and therapeutic properties of plants and their bioactive components, the medical sector paying great attention to their possible advantages (Saghir et al., 2016). *Nigella sativa* (*N. sativa*) is a popular medicinal herb used to cure a variety of ailments (Saghir et al., 2021). Thymoquinone (THY) is the most prominent bioactive component in *N. sativa* seed essential oil. It has shown to possess a range of actions, including antibacterial (Hosseinzadeh et al., 2012) antioxidants (Hosseinzadeh et al., 2012) anti-inflammation (Bai et al., 2014), hepatoprotective (Aycan et al., 2014), and anticancer properties (Talib and AbuKhader, 2013). Additionally, the use of nanotechnology techniques improved the management of numerous human diseases due to its improved bioavailability and drug delivery (El-Far et al., 2018). One of the most significant benefits of nano-nutraceuticals is their capacity to decrease renal clearance for a prolonged TQ pharmacological impact. They can also readily cross biological membranes and deliver sustained TQ release to various parts of the body (Srinivas et al., 2010).

As a result, the current study intends to evaluate and sort out the impact of IL-35 and thymoquinone-nanoparticles (THYO) on PAR-induced hepatotoxicity rats and oxidative stress, as well as exploring the immunological, apoptotic, and inflammatory cytokine responses.

## 2 Materials and methods

### 2.1 Chemicals

Interleukin-35 (IL-35) recombinant protein (MBS2011667) was supplied by MyBioSource Company (San Diego, USA). Thymoquinone (24856596) was acquired from Sigma-Aldrich Com (Taufkirchen Germany).

### 2.2 Animals and experimental design

Sixty male Swiss rats (180–200 g), were housed in a closely monitored environment (temperature: 23.5 ± 2 °C; relative humidity: 56 ± 8 %; and sustained on a 12 h dark/light cycles). The rats were provided with water and food *ad libitum*. Subsequent a week of adaptation, rats were organized into six groups (n = 10) as illustrated in Supplementary Fig. 1. Group I (control group) administered orally 0.5 mL normal saline, while groups 2 and 3 received IL-35 (200 ng/kg) (Wu et al., 2016) and TYHO (0.5 mg/kg) intraperitoneally (i.p) (Darakhshan et al., 2015), respectively. Group 4 administered PAR (600 mg/kg orally, p.o), while groups 5 and 6 were received PAR (600 mg/kg) and treated with IL-35 (200 ng/kg) and TYHO (0.5 mg/kg) for 21 days, respectively.

**Fig. 1:**
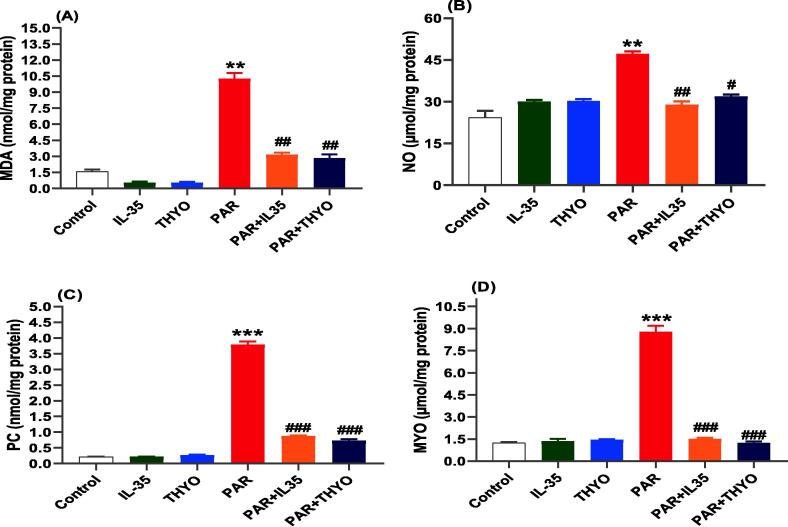
Hepatic oxidative damage induced by PAR in rats was inhibited by IL-35 and THYO. Changes in malondialdehyde (MDA) (A), nitric oxide (NO) (B), protein carbonyl (PC) (C), and myeloperoxidase (MYO) (D) in control group (Control), (IL-35); rats received IL-35 (200 ng/kg), THYO; rats received THYO (0.5 mg/kg), PAR; rats received PAR (600 mg/kg), PAR + IL-35; rats received PAR (600 mg/kg) and treated with IL-35 (200 ng/kg), PAR + TYHO; rats received PAR (600 mg/kg) and treated with THYO (0.5 mg/kg). Data were presented as mean ± SEM. One-way ANOVA followed by Tukey's post-hoc tests were used for comparing data at significance level *p* < 0.05. *p < 0.05, **p < 0.01 and ***p < 0.01 indicate changes in PAR induced group compared to control group, whereas #*p <* 0.05, ##*p <* 0.01 and ###*p <* 0.001 show changes in groups treated with IL-35 or THYO against untreated PAR-induced group.

TYHO synthetization was conducted using the double emulsion method of TYHO encapsulation into poly lactic-co-glycolic acid (PLGA) as asserted early (Al-Gabri et al., 2019). The protocol received approval from the Ethics of Animal Use in Research Committee of the Faculty of Veterinary Medicine, Badr University in Cairo (BCU-IACUC/VET) (Approval number, BUC-IACUC/VET/130/A/2023) and the research methods were carried out in accordance with NIH criteria. Every effort was made to ensure the humane handling of the animals and address ethical considerations throughout the study.

### 2.3 Blood and tissues sampling

On day 22, animals were deprived of food overnight, weight and blood specimens were acquired via the *retro*-orbital plexus site in two tubes; one containing EDTA for hematological assay and the other non-heparinized tubes for serum collection. Serum was split using a centrifuge for 10 min at 1500 g and reserved at 20 °C for subsequent exploration. Then, xylazine and ketamine (10 and 100 mg/kg/b.wt, respectively) via intramuscular injection were used to anesthetize rats, executed by cervical dislocation, hepatic tissues were harvested and washed by PBS in sterilized cups. Direct immersion of liver tissues in buffered formalin (10%) was done for subsequent histological and immunohistochemical evaluation. Additional liver samples were dissolved in cold PBS before being centrifuged. The resulting supernatant was stowed at −80 °C.

### 2.4 Assessment of hematology and biochemical markers

Parameters for complete blood count (CBC) were assessed using hematology analyzer (Hospitex Diagnostic, Italy) (Bain et al., 2016). Biochemical markers such as total protein, albumin and glucose levels were assessed using kits from Bio-diagnostic, Giza, Egypt (Wang et al., 2010). Globulin levels were detected by subtracting albumin from the total protein, and A/G values were calculated by dividing albumin to globulin. According to the provided instructions, total bilirubin (MBS730053) and direct bilirubin (MBS1607953) were assessed using ELISA kits (MyBioSource, San Diego, USA). Liver enzymes including aspartate transaminase (AST), alanine transaminase (ALT), alkaline phosphatase (ALKP), gamma glutamyl transferase (γGT) and lactate dehydrogenase (LDH) were measured using kits (Bio-diagnostic) (Murray and Kaplan, 1984, Burtis and Ashwood, 1994). In addition, total cholesterol (TC), triglycerides (TG), in addition to high-density lipoprotein cholesterol (HDL-C), and low-density lipoprotein cholesterol (HDL-C) measurements were taken using Roche Cobas 8000 analyzer system (Mannheim, Germany).

### 2.5 Oxidative stress assessment

Liver homogenates were produced and utilized to determine the antioxidant enzymes activities of catalase (CA) CA2517 (Aebi, 1984), superoxide dismutase (SOD) SD2521 (Nishikimi et al., 1972), reduced glutathione (GSH) GP2524 (Alía et al., 2003), besides total antioxidant capacity (TAC) TA2526 (Alía et al., 2003). The levels of protein carbonyl (PC) PC2530 (Davies et al., 2001), malondialdehyde (MDA) MD2529 (Uchiyama and Mihara, 1978) and myeloperoxidase (MYO; MY2531 (Hillegass et al., 1990) were determined following the instructions provided in the kits (Bio-diagnostic, Giza, Egypt).

### 2.6 Assessment of immunological markers and hepatic inflammatory response

Serum lysozyme activity was determined using rat ELISA kit (ab211113) acquired form Abcam Company (Abcam company, Cambridge CB20AX, UK) according to the procedure of Akinbi methos (Akinbi et al., 2000). Using the Griess reagent method, nitric oxide was performed following the Rajaraman method (Rajaraman et al., 1998). Immunoglobulin M (IgM; MBS700907), and immunoglobulin G (IgG; MBS2513365) were determined via ELISA kits (MyBio Source, San Diego, USA).

Also, tumor necrosis factor alpha (TNF-α) (E-EL-R2856), nuclear factor kappa B (NF-κB) (E-EL-R0674), interleukins 10 (IL-10) (E-EL-R0016), interleukins 6 (IL-6) (E-EL-R0015), γ interferon (IFN-γ) (E-EL-R0009), cytochrome *C* (cyt C) (E-EL-R0006), pro-apoptotic caspase-3 (CAS-3) (E-EL-R0160), and Bax were assayed in the hepatic tissues using specific ELISA kits supplied by Elabscience Company (Texas, 77079, USA) (USA), whereas anti-apoptotic (Bcl-2) was determined using ELISA kit (MBS2512405) supplied form MyBioSource (USA).

### 2.7 Histological study

Liver tissues specimens were preserved for 48 h in natural buffered formalin (10%), dehydrated in ethanol (70–100%), then xylene was used to clear, and lastly encased in paraffin wax. About 5 μm thick of paraffin units were obtained by using automated microtome. Then, Hematoxylin and Eosin dyes were used for staining (Suvarna et al., 2018). A scoring system was used based on the examination of five fields in each slide as the follows: (-=No changes 0%, 1+=mildly 15–25%, 2+=moderate 30–65%, 3+=severe upto70%) according to previous syudies (Bedossa et al., 2012, Kleiner et al., 2005).

### 2.8 Liver immunohistochemistry

Immunohistochemistry (IHC) was used to evaluate changes in TNF-α, NF-κB p65, and PCNA in hepatic tissues as descried previously (Hsu, et al., 1981). Briefly, 5 μm sections were dewaxed, and retrieved using citrate buffer (pH 6.8). Following that, endogenous peroxidase enzyme was blocked thru hydrogen peroxide (0.3%) in the processed tissue slices. The tissues were then probed with anti-TNF-α (dilution 1:1000) (ab307164, Abcam, USA), anti-NF-κB p65 (dilution 1:200) (E-AB-22066; Elabscience Company, USA), and anti-PCNA (dilution 1:50) (ab92729; Abcam, USA). After an overnight incubation at 4 °C, the plates were rinsed by PBS. Then, incubated with the selected antibody for 30 min at 22–25 °C. Visualization of the slides was achieved using a DAB kit, and counterstaining was performed with Mayer's hematoxylin (Elnagar et al., 2021). The intensity score of immunohistochemically markers were determined with Image J software analysis (NIH, USA) following the previous research (Nishad et al., 2023).

### 2.9 Extraction of total RNA and real time-PCR

Hepatic inflammatory related genes including TNF-α, TGF-β, IL1β, IL10, Bax, Bcl2 and Casps-3 was examined using qRT-PCR as early described (Al-Shahat et al., 2022). Extraction of RNA from hepatic tissues was achieved using Trizol reagent (Invitrogen; USA). Following the extraction, the quality of RNA was evaluated by a NanoDrop® ND-1000 Spectrophotometer (NanoDrop Technologies, Delaware, USA). Subsequently, synthesis of cDNA was achieved through using the HiSenScript™ RH (-) cDNA Synthesis kit (iNtRON Biotechnology Co., South Korea) (Al-Shahat et al., 2022, Saber et al., 2022). Analysis of gene expression was conducted using qRT-PCR (Agilent Stratagene, USA) by 5x HOT FIRE Pol EvaGreen qPCR Mix Plus (Solis BioDyne, Tartu, Estonia), according to supplier's directions. The PCR cycling conditions were determined as follows; primary denaturation (15 min at 95 °C), 40 cycles of denaturation (30 s at 95 °C), annealing (60 s at 60 °C, and extension (60 s at 72 °C). The designed primers using Primer-BLAST (NCBI) with forward and reverse sequences and size are demonstrated in Supplementary Table 1 (Arisha et al., 2019, Al-Shahat et al., 2022). The collected findings were assessed using the 2^-ΔΔCt^ method and standardized to the GAPDH gene (Livak and Schmittgen, 2001).

### 2.10 Statistical assessment

Findings were presented as mean ± SEM. Graph-Pad software version 9.0 and SPSS (version 21) were used for data analysis. One-way ANOVA followed by Tukey's post-hoc tests were used for comparing data at significance level *p* < 0.05. *p < 0.05, **p < 0.01 and ***p < 0.01 indicate changes in PAR induced group compared to control group, whereas ^#^*p <* 0.05, ^##^*p <* 0.01 and ^###^*p <* 0.001 show changes in groups treated with IL-35 or THYO against untreated PAR-induced group.

## 3 Results

### 3.1 Impact of IL-35 and THYO on hematological indices

The effects of PAR- induced hepatic toxicity and its co-treatment with IL-35 or THYO on hematology parameters are presented in Supplementary Table 2. The findings did not exhibit any discernible variations in the mean values between control group and groups treated with only IL-35 or THYO.

The PAR treated group revealed a significant declines in RBC, Hb, PCV, WBC (*p <* 0.05) and MCV (*p <* 0.01) compared to normal group. In addition, both IL-35 and THYO groups demonstrated statistically significant increases in the mean values of RBC (*p <* 0.05), Hb (*p <* 0.01), PCV, MCV, and WBC (*p <* 0.05) versus PAR control group. Other parameters such as MCH, MCHC, PLT, and differential counts of WBC showed no statistically significant changes (Supplementary Table 2).

### 3.2 IL-35 and THYO mitigate PAR-induced liver injury through restoring the biochemical parameters to their normal values

It was depicted in Table 1 that PAR caused substantial rises (*p <* 0.001) of liver function tests (total, direct and indirect bilirubin, ALT, AST, ALP, GGT, and LDH) in serum when contrasting with control group. Nevertheless, therapy with IL-35 and THY individually did not result in any significant alterations in liver function tests juxtaposed with control group. The PAR group injected with IL-35 had significantly lower levels of total, direct, indirect bilirubin ALT, AST, ALP, GGT, and LDH (*p <* 0.001) (Table 1) in contrast with untreated PAR group. At the same time, PAR group treated with THYO demonstrated a significant decrease in levels of hepatic parameters, compared with untreated PAR group. Notably, neither of the two groups administered with IL-35 or THYO alone had any significant differences in all variables studied compared to control group. Treatment with IL-35 or THYO dramatically reduced liver damage, as seen by the improvement of the elevated serum AST, ALP, ALT, and LDH levels (Table 1). Table 1 further demonstrates that as compared to the normal control group, PAR significantly decreased the amounts of total protein, albumin, and globulin. However, giving IL-35 or THYO to the PAR-induced groups exhibited statistically significant recovers of total protein, globulin as well as albumin (*p <* 0.05), in parallel with a significant increases in A/G ratio (*p <* 0.05) for both of IL-35 or THYO groups.

**Table 1:** Liver injury induced by PAR exhibited statistical changes in the biochemical parameters which were alleviated by IL-35 and THYO.

**Test**	**Control**	**IL-35**	**THYO**	**PAR**	**PAR + IL-35**	**PAR + THYO**
T.bilirubin	0.56 ± 0.03	0.51 ± 0.07	0.43 ± 0.02	3.09 ± 0.38***	0.75 ± 0.17^###^	0.51 ± 0.10^###^
D.bilirubin	0.15 ± 0.01	0.14 ± 0.01	0.12 ± 0.02	1.81 ± 0.22***	0.16 ± 0.02^###^	0.13 ± 0.02^###^
Ind.bilirubin	0.42 ± 0.01	0.37 ± 0.08	0.43 ± 0.10	1.28 ± 0.17***	0.56 ± 0.13^##^	0.38 ± 0.09^###^
AST	123.40 ± 9.61	107.35 ± 10.61	90.06 ± 1.66	339.73 ± 15.67***	141.80 ± 3.75^###^	109.83 ± 12.03^###^
ALT	45.77 ± 1.04	43.96 ± 1.49	43.61 ± 2.40	245.21 ± 5.63***	55.904.44^###^	59.63 ± 4.04^###^
ALKP	13.67 ± 0.72	16.68 ± 0.88	17.13 ± 0.33	117.54 ± 3.85***	14.25 ± 0.81^###^	15.06 ± 0.77^###^
γGT	1.78 ± 0.08	1.37 ± 0.16	1.26 ± 0.05	2.90 ± 0.07***	1.54 ± 0.04^###^	1.74 ± 0.16^##^
LDH	364.13 ± 10.4	263.33 ± 3.71	235.67 ± 4.10	52.00 ± 11.28^***^	154.10 ± 6.01^###^	224.00 ± 36.00^##^
T.protein	3.20 ± 0.07	2.81 ± 0.11	11.00 ± 8.20	3 ± 0.09**	3.08 ± 0.21^#^	3.38 ± 0.10^#^
Albumin	1.37 ± 0.09	1.46 ± 0.16	1.65 ± 0.14	1.29 ± 0.02*	1.44 ± 0.18^#^	1.24 ± 0.04^#^
Globulin	1.58 ± 0.21	1.35 ± 0.12	1.46 ± 0.03	1.72 ± 0.06*	1.64 ± 0.04^#^	2.14 ± 0.07^#^
A/G ratio	0.85 ± 0.12	1.11 ± 0.19	1.13 ± 0.08	0.79 ± 0.04**	0.84 ± 0.06^#^	0.58 ± 0.02^#^
TC	89.00 ± 1.21	90.74 ± 1.33	89.38 ± 1.36	129.28 ± 5.25***	94.52 ± 2.27^##^	93.27 ± 1.01^##^
TG	66.99 ± 2.53	67.96 ± 1.10	69.30 ± 2.63	140.81 ± 5.62***	68.76 ± 0.95^###^	71.86 ± 2.87^###^
HDL-C	48.13 ± 0.97	49.95 ± 0.94	49.40 ± 0.64	148.20 ± 1.10***	51.22 ± 0.53^#^	47.78 ± 0.71^#^
LDL-C	53.48 ± 2.97	30.53 ± 3.90	49.50 ± 1.30	18.97 ± 1.00***	66.73 ± 1.44^##^	42.79 ± 6.28^##^
VLDL-C	10.75 ± 1.08	10.59 ± 0.79	9.68 ± 0.41	26.73 ± 1.32***	14.40 ± 0.51^##^	14.37 ± 0.57^##^

T.bilirubin; total bilirubin, D. bilirubin; direct bilirubin, Ind. Bilirubin; indirect bilirubin, AST; aspartate transaminase, ALT; alanine transaminase, ALKP; alkaline phosphatase, γGT; gamma glutamyl transferase, LDH; lactate dehydrogenase, T. protein; total protein, A/G ratio: albumin/globulin, TC; total cholesterol, TG; triglycerides, HDL-C; high density lipoprotein cholesterol, LDL-C; low density lipoprotein cholesterol, VLDL-C; very low density lipoprotein. Data were presented as mean ± SEM. One-way ANOVA followed by Tukey's post-hoc tests were used for comparing data at significance level *p* < 0.05. *p < 0.05, **p < 0.01 and ***p < 0.01 indicate changes in PAR induced group compared to control group, whereas #*p <* 0.05, ##*p <* 0.01 and ###*p <* 0.001 show changes in groups treated with IL-35 or THYO against untreated PAR-induced group.

Lipid markers including TG, TC, VLDL-C and LDL-C exhibited a substantial rise in PAR-induced group against control group (*p <* 0.001), while HDL-C showed significant decreases in comparison with PAR induced group (*p <* 0.001). There were no substantial modifications in lipid profile markers between the two groups given IL-35 or THYO alone and the control group. As compared to the PAR-induced group, treatment with IL-35 and THYO improved lipid profile parameters, returning them to lower values, and showed statistically significant declines for TC, TG, LDL-C, and VLDL-C (*p <* 0.01). Treatment with IL-35 and THYO simultaneously pointedly improved HDL-C levels compared to the PAR group (*p <* 0.05) (Table 1).

### 3.3 IL-35 and THYO mitigate oxidative stress

**Fig. 2:**
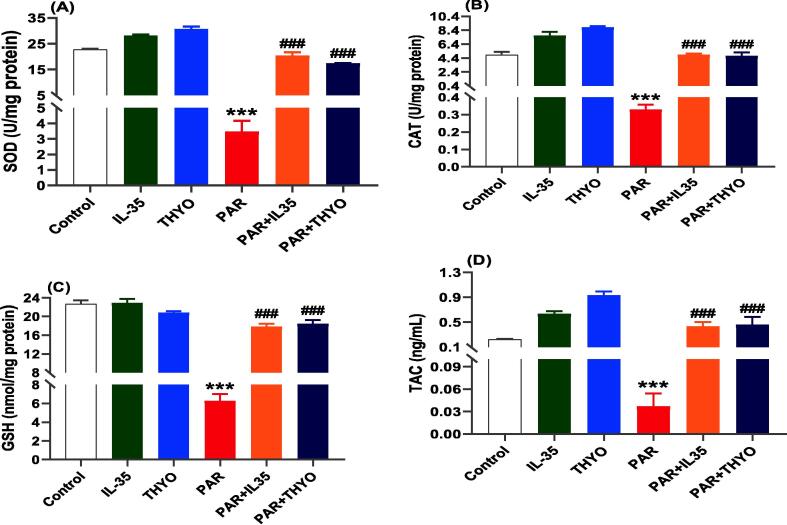
Hepatic oxidative damage induced by PAR in rats was restored by IL-35 or THYO. Changes in oxidative damage parameters including SOD (A), CAT (B), GSH (C), and TAC (D) in control group (Control), (IL-35); rats received IL-35 (200 ng/kg), THYO; rats received THYO (0.5 mg/kg), PAR; rats received PAR (600 mg/kg), PAR + IL-35; rats received PAR (600 mg/kg) and treated with IL-35 (200 ng/kg), PAR + TYHO; rats received PAR (600 mg/kg) and treated with THYO (0.5 mg/kg). Data were presented as mean ± SEM. One-way ANOVA followed by Tukey's post-hoc tests were used for comparing data at significance level *p* < 0.05. *p < 0.05, **p < 0.01 and ***p < 0.01 indicate changes in PAR induced group compared to control group, whereas #*p <* 0.05, ##*p <* 0.01 and ###*p <* 0.001 show changes in groups treated with IL-35 or THYO against untreated PAR-induced group.

Oxidative stress is one of the crucial pathological processes that could be occured as a result of PAR-induced liver damage. PAR treated group showed significant increases in hepatic markers, including MDA, NO, PC, and MYO (*p* < 0.001). The administration of IL-35 or THYO to PAR-induced rats resulted in significant decreases in antioxidants parameters compared with untreated PAR-induced group (Fig. 1A-D). Whereas, as demonstrated in Fig. 2, the antioxidant levels of rats given only IL-35 or THYO are comparable with control group. Conversely, PAR triggered markedly diminution in SOD, CAT, GSH, and TAC, which was restored by IL-35 or THYO as depicted in Fig. 2A-D.

### 3.4 Effects of IL-35 and THYO on immunoglobulins

**Fig. 3:**
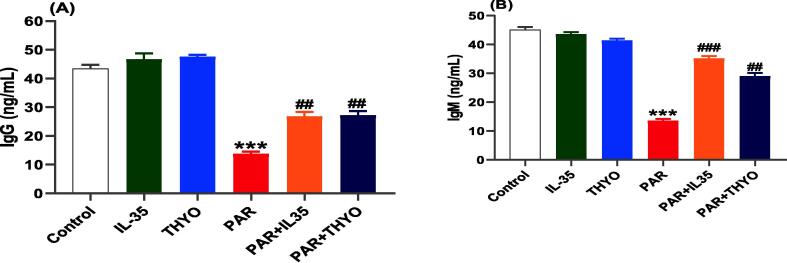
Inhibition of immune markers by PAR in rats was restored by IL-35 or THYO. Changes in IgG (A) and IgM (B) in control group (Control), (IL-35); rats received IL-35 (200 ng/kg), THYO; rats received THYO (0.5 mg/kg, i.p), PAR; rats received PAR (600 mg/kg), PAR + IL-35; rats received PAR (600 mg/kg) and treated with IL-35 (200 ng/kg), PAR + TYHO; rats received PAR (600 mg/kg) and treated with THYO (0.5 mg/kg). Data were presented as mean ± SEM. One-way ANOVA followed by Tukey's post-hoc tests were used for comparing data at significance level *p* < 0.05. *p < 0.05, **p < 0.01 and ***p < 0.01 indicate changes in PAR induced group compared to control group, whereas #*p <* 0.05, ##*p <* 0.01 and ###*p <* 0.001 show changes in groups treated with IL-35 or THYO against untreated PAR-induced group.

By measuring levels of IgG and IgM, the immune-protective effect of IL35 or THYO against PAR toxicity in rats was evaluated as presented in Fig. 3. PAR exposure resulted in a considerable drop of IgM and IgG levels in contrast to control group. After treatment with IL-35 or THYO, a significant increase was exhibited for both IgG (*p <* 0.01) and IgM (*p <* 0.001, and *p <* 0.01, respectively) compared with PAR control group. Both IL35 and THYO group had higher levels of IgG and IgM; however these elevations were not significant compared to the control.

### 3.5 IL-35 and THYO alleviate hepatic inflammation

**Fig. 4:**
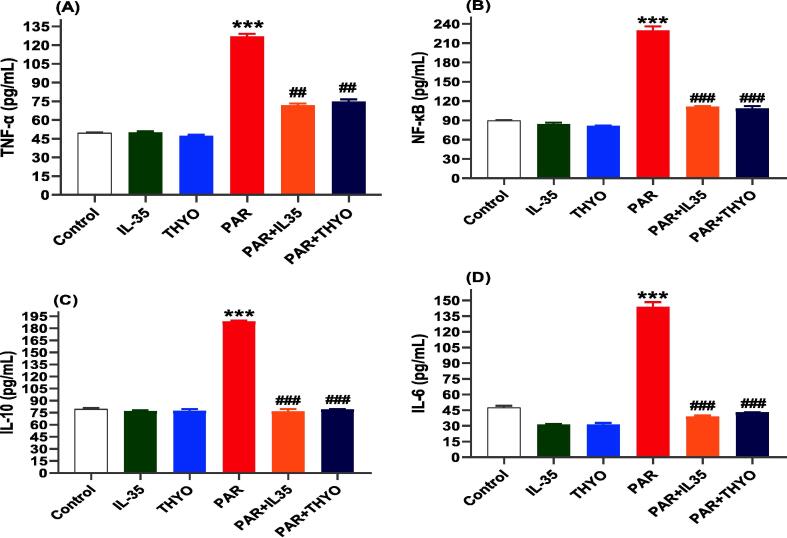
Hepatic inflammation in PAR-induced rats was attenuated by IL-35 or THYO. Changes in NF-κB (A), TNF-α (B), IL-10 (C), and IL-6 (D) in control group (Control), (IL-35); rats received IL-35 (200 ng/kg), THYO; rats received THYO (0.5 mg/kg), PAR; rats received PAR (600 mg/kg), PAR + IL-35; rats received PAR (600 mg/kg) and treated with IL-35 (200 ng/kg), PAR + TYHO; rats received PAR (600 mg/kg) and treated with THYO (0.5 mg/kg). Data were presented as mean ± SEM. One-way ANOVA followed by Tukey's post-hoc tests were used for comparing data at significance level *p* < 0.05. *p < 0.05, **p < 0.01 and ***p < 0.01 indicate changes in PAR induced group compared to control group, whereas #*p <* 0.05, ##*p <* 0.01 and ###*p <* 0.001 show changes in groups treated with IL-35 or THYO against untreated PAR-induced group.

In the current investigation, we assessed the effects of IL-35 and THYO on inflammatory responses, including activation of NF-κB and generation of pro-inflammatory cytokines, by measuring levels of NF-κB, TNF-α, IL-10, and IL-6, since these indicators are key mediators in the process. Herein, PAR persuaded an enormous increase in hepatic NF-κB and levels of TNF-α, IL-6, and IL-10 in the liver compared with the normal control (Fig. 4A-D). Treatment with IL-35 and THYO significantly reduced the levels of NF-κB, TNF-α, IL-10, (*p* < 0.01) and IL-6 (*p* < 0.001) when compared to PAR group. However, THYO or IL35 alone did not significantly affect these factors in healthy rats (*p* > 0.05) (Fig. 4A-D).

### 3.6 Effects of IL-35 and THYO on apoptotic and mitochondrial markers

**Fig. 5:**
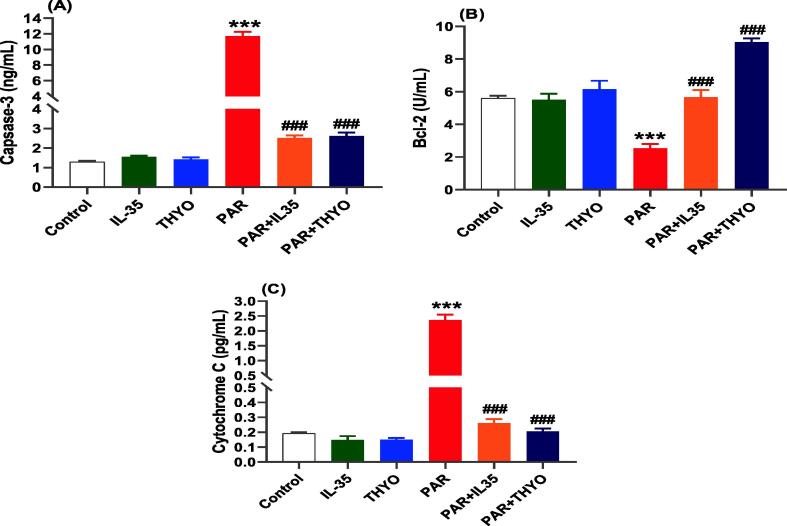
Effects of IL-35 and THYO on the levels of apoptosis and mitochondrial proteins expressed in the liver. Changes in Caspase-3 (A), Bcl-2 (B) and cytochrome *c* (C) in control group (Control), (IL-35); rats received IL-35 (200 ng/kg), THYO; rats received THYO (0.5 mg/kg), PAR; rats received paracetamol (600 mg/kg), PAR + IL-35; rats received PAR (600 mg/kg) and given IL-35 (200 ng/kg), PAR + TYHO; rats received PAR (600 mg/kg) and treated with THYO (0.5 mg/kg). Data were presented as mean ± SEM. One-way ANOVA followed by Tukey's post-hoc tests were used for comparing data at significance level *p* < 0.05. *p < 0.05, **p < 0.01 and ***p < 0.01 indicate changes in PAR induced group compared to control group, whereas #*p <* 0.05, ##*p <* 0.01 and ###*p <* 0.001 show changes in groups treated with IL-35 or THYO against untreated PAR-induced group.

Hepatic expression levels of apoptosis and mitochondrial regulatory proteins; such as caspase-3, Bcl-2 and cytochrome *C* were examined further to investigate the impact of IL-35 and THYO on PAR-induced liver damage rats (Fig. 5). As shown in Fig. 5, apoptotic indicators caspase-3 and the mitochondrial marker cytochrome *c* were significantly higher in PAR than in normal control group, whereas levels of Bcl-2 was markedly reduced. On treatment with IL-35 or THYO, significant reduction was noticed for caspase-3 and cytochrome *c*, while significant increase was detected for Bcl-2 in contrast with the PAR induced group. In healthy rats, THYO or IL-35 treatment alone had no effect on the hepatic apoptosis or mitochondrial markers.

### 3.7 Effects of IL-35 and THYO on the expression of genes associated with liver injury

**Fig. 6:**
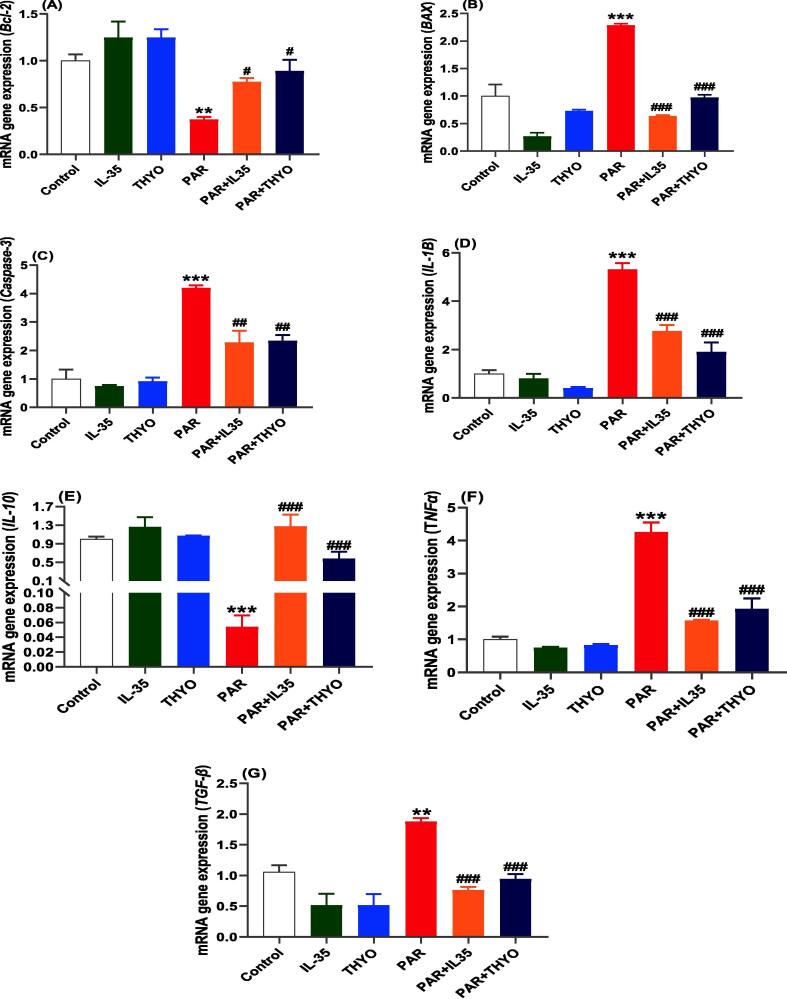
IL-35 and THYO upregulated Bcl-2 and IL-10 and downregulated Bax, caspase-3, IL-1β, TNF-α, and TGF-β genes expression in PAR control group. Changes in Bcl-2 (A), Bax (B), caspase-3 (C), IL-1β (D), IL-10 (E), TNF-α (F), and TGF-β (G) in control group (Control), (IL-35); rats received IL-35 (200 ng/kg), THYO; rats received THYO (0.5 mg/kg), PAR; rats received PAR (600 mg/kg), PAR + IL-35; rats received PAR (600 mg/kg) and treated with IL-35 (200 ng/kg), PAR + TYHO; rats received PAR (600 mg/kg) and treated with THYO (0.5 mg/kg). Data were presented as mean ± SEM. One-way ANOVA followed by Tukey's post-hoc tests were used for comparing data at significance level *p* < 0.05. *p < 0.05, **p < 0.01 and ***p < 0.01 indicate changes in PAR induced group compared to control group, whereas #*p <* 0.05, ##*p <* 0.01 and ###*p <* 0.001 show changes in groups treated with IL-35 or THYO against untreated PAR-induced group.

To confirm our findings, we assessed the expression of Bcl-2, Bax, caspase-3, IL-1β, IL-10, TNF-α, and TGF-β genes implicated in molecular pathways (Fig. 6). In compared to the control group, there were no disparities in hepatic Bcl-2 gene expression between groups given IL-35 or THYO alone. In contrast, the PAR-induced group considerably downregulated the Bcl-2 and IL-19 genes compared to the control group, but the IL-35 or THYO-treated groups significantly increased Bcl-2 and IL-10 expression opposed to the PAR-induced group (Fig. 6A, E). Moreover, PAR induced significant upregulations in the Bax, caspase-3, IL-1β, TNF-α, and TGF-β genes, which were significantly downregulated by IL-35 or THYO (Fig. 6B, C, D, F, G). Overall, IL-35 and THYO have improved the pro-inflammatory genes in rats.

### 3.8 Effects of IL-35 and THYO on the histopathological findings on PAR-induced liver injury rats

**Fig. 7:**
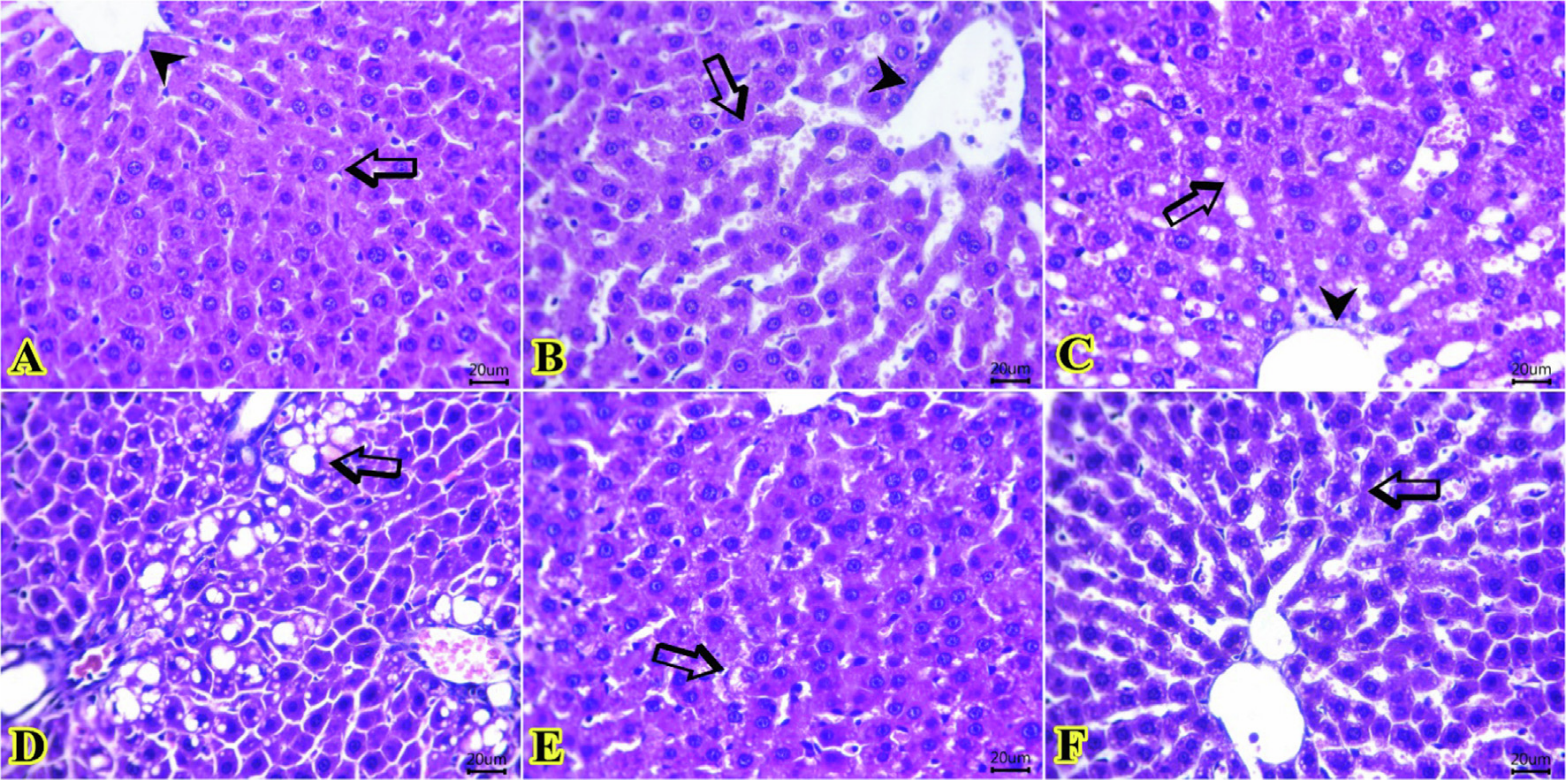
Photomicrograph of H&E stained sections from liver (20 μm) showing: normal histological structures of hepatic cords (arrow), sinusoids, and central vein (arrowhead) in negative control group (A), IL-35 only group (B), and THYO only group (C). Areas of periportal steatosis (arrow), and congested of hepatic vasculatures in PAR-induced group (D). Apparently normal with a few hydropic degenerated cells (arrow) in treated toxic group with PAR + IL-35 (E). Minute droplets of fatty globules in a moderate area of hepatic lobules (arrow) in treated toxic group with PAR + THYO (F).

The histopathological evolution of the liver tissues from negative control group (Fig. 7A) showed normal histo-morphological structures of hepatic lobs, lobules, hepatic cords, sinusoids, portal triads (portal vein, artery and bile duct), and central veins. Similarly, both treated group with IL-35 only ((Fig. 7B) and THYO only ((Fig. 7C) showed preserved hepatocytes arrangement, sinusoids, central veins and portal areas. In contrast, the PAR-treated group (Fig. 7D) exhibited marked periportal micro and macro steatosis (fatty liver) with some coalescing to form fat sacs. In addition, prominent widening sinusoids, congested hepatic blood vessels and sinusoids with compensatory atrophied hepatic cords (hypotrophy hepatocytes) were observed. Individuals/multifocal necrotic areas were observed, which particularly replaced by inflammatory cell aggregates. However, in the group of rats with PAR toxicity and treated with IL-35 (Fig. 7E), restoration of the tissues histological structure to the normal except for mild to moderate hepatocytic hydropic degeneration were demonstrated. Prominent diploneuclei hepatocytes, indicative of regeneration, were also observed. Moreover, rats with PAR toxicity and treated with THYO ((Fig. 7F) displayed minute droplets of fatty globules at centrilobular and mid zonal areas in a few numbers of hepatic lobules but the remaining areas showed normally appearance (Table 2).

**Table 2:** Score of the histopathological changes and regenerative indexes in five sections in each group.

Criteria	Control	IL-35	THYO	PAR	PAR + IL-35	PAR + THYO
Hepatitis	–	–	–	3+	–	–
Necrosis	–	–	–	2+	–	–
Steatosis	–	–	–	3+	1+	–
Congestions	–	–	–	3+	1+	1+
Hepatocytes degenerations	–	–	–	2+	3+	1+
Apoptosis	–	–	–	3+	1+	1+
Widening sinusoids	–	–	1+	3+	1+	2+
Diploneuclei hepatocytes	–	1+	1+	1+	2+	3+
Regenerative changes	–	1+	1+	–	3+	3+

Score system (-=No changes 0%, 1+=Mildly 15–25%, 2+=Moderate 30–65%, 3 + Severe upto70%).

### 3.9 IL-35 and THYO ameliorate and downregulate the expression of TNF-α, PCNA and NF-κB on PAR-induced liver injury rats

**Fig. 8:**
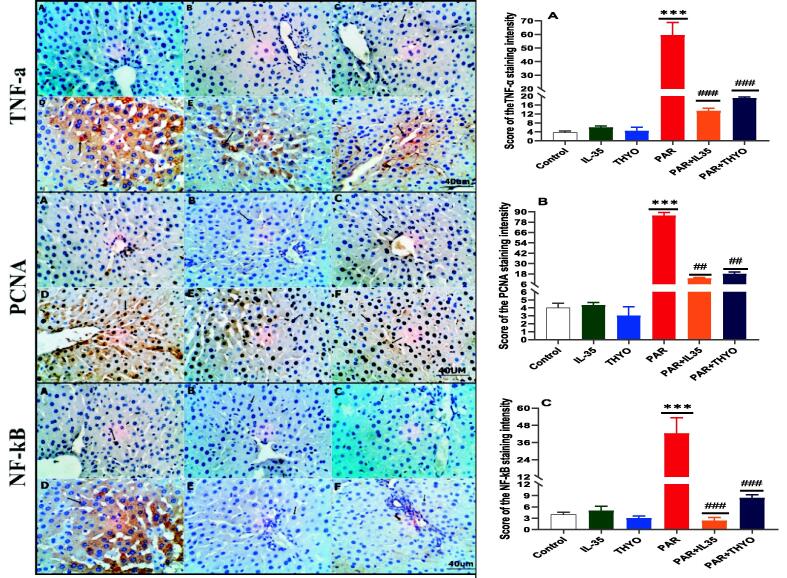
Representative photomicrograph of the TNF-α, PCNA and Nf-kB IHC markers showing absence of immunoreactivity stain deposits in control (A), IL-35 (B) and THYO (C) groups. While, strong intensity of immune-reactivity was observed as a result of increase expressions in PAR-treated groups (D). Moreover, remodelling of the markers expression appeared as mildly immunoreactivity in both PAR + IL35 (E) and PAR + THYO (F) treated groups (Scale bar 40 μm). Vertical columns on the right charts of the represent mean, while error bars represent standard deviation. IHC: TNF-α (A), PCNA (B) and NF-kB (C). Control group (Control), (IL-35); rats received IL-35 (200 ng/kg), THYO; rats received THYO (0.5 mg/kg), PAR; rats received paracetamol (600 mg/kg), PAR + IL-35; rats received PAR (600 mg/kg) and treated with IL-35 (200 ng/kg), PAR + TYHO; rats received PAR (600 mg/kg) and treated with THYO (0.5 mg/kg). Data were presented as mean ± SEM. One-way ANOVA followed by Tukey's post-hoc tests were used for comparing data at significance level *p* < 0.05. ***p < 0.01 indicate changes in PAR induced group compared to control group, whereas ##*p <* 0.01 and ###*p <* 0.001 show changes in groups treated with IL-35 or THYO against untreated PAR-induced group.

As depicted in Fig. 8, the TNF-α, PCNA and NF-κB immune-depression was disappearing the brownish deposition stain due to negative immune-reactive of immune-marker in control, IL-35 and THYO only treated rats. In contrast, the PAR induced-rats revealed strong intensity of immunoreactivity du to increase expression of TNF-α, PCNA and NF-κB immune-markers. On the other hands, the rats treated with PAR + IL35 and PAR + THYO showed downregulation of the expression of TNF-α, PCNA and NF-κB, respectively compared to the PAR group. The intensity scores systems were analyzed with ImageJ software and presented on the right side of Fig. 8. A significant increase was detected in PAR-induced group in comparison to normal control group (*p <* 0.001), while a substantial reduction was perceived in PAR-treated groups with IL-35 and THYO compared to PAR-induced group (*p <* 0.001).

## 4 Discussion

Liver disease is one of the most serious aliments in the world, with high incidence and mortality rate, and its prevalence is growing yearly (Zhang et al., 2021). Chronic inflammation and oxidative stress are the main causes of liver disease, which can result in the assembly of an extracellular matrix that could lead to hepatitis, fibrosis, cirrhosis, and hepatocellular cancer (Hu et al., 2021). Currently, there are limited effective options for treating liver illnesses. Therefore, it is crucial to enhance our understanding of the underlying mechanisms of liver damage in order to develop more potent drugs for prevention and treatment of liver diseases (Omenesa Bello et al., 2018, Islam et al., 2021).

IL-35, a recently identified cytokine, has been shown to shield liver against environmental assault through boosting the percentage of Treg, which can produce anti-inflammatory cytokines and limit immune cellular growth (Hu et al., 2021). As previously stated, more research is needed to completely comprehend the role of IL-35. To date, few studies have been undertaken to investigate the detailed activities of IL-35 in preventing and suppressing variety of diseases. In addition, THY is one of the prevalent phytochemicals with potential biological activities, particularly antioxidant, anti-inflammatory, antibacterial, and anticancer properties, which make it a promising treatment for a range of diseases. Furthermore, one of the noteworthy aspects of IL-35 is its capacity to limit excessive autoimmune response by reducing Th1 and Th17 development. This prevents liver from developing immunity to chronic infections brought on by viral damage (Yang et al., 2020b). As a result, we thoroughly evaluated and compared the effects of IL-35 and THYO on hematological, biochemical, and immunological parameters, along with apoptosis, gene expression, histological, and immunohistochemical assessments.

PAR is proved as an effective hepatotoxin that is broadly utilized in different cellular and tissues models as a liver injury inducer (Akther et al., 2013). The degree of hepatic damage has been closely connected with reduction in oxidative stress indicators, including CAT, SOD, LPO, and GSH. In parallel, hepatotoxicity is evidenced by the rise in AST, ALT, ALP enzymes, and bilirubin because these enzymes are found in the cytoplasm and are released into the blood due to cell damage (Agarwal and Ghobrial, 2013, Gutiérrez and Solís, 2009). Using of PAR produced similar findings which are in agreement with prior recorded outcomes (Barros et al., 2017, Islam et al., 2021).

The results of the existing study demonstrated that PAR causes a decline in RBC and Hb, an increase in hepatic oxidative damage, inflammation, and apoptosis. PAR could induce progression of fatty liver, widening sinusoids, congested hepatic blood vessels, and sinusoids with compensatory atrophied hepatic cords and hypotrophy inflammatory cell aggregates, which are the main driving factors behind PAR hepatotoxicity (Fortea et al., 2020).

Also, in the present study, compared to the untreated PAR group, treatments for three weeks with IL-35 or THYO demonstrate their ability to recover RBC, Hb, and WBC to normal levels. Transaminases ALT and AST are commonly used as an indication of hepatic necrosis because they are associated with the reductive conversion of amino acids to form pyruvate or oxalate, respectively. When hepatocytes are damaged, ALT and AST are released into the extracellular environment. Consequently, an increase in these enzymes is believed to be a marker of liver injury (Contreras-Zentella and Hernández-Muñoz, 2016).

In this regards, a substantial improvement in liver function indicators was noticed, which explains the capacity of both IL-35 and THYO to alleviate the effects caused by PAR and restore bilirubin and liver enzymes AST, ALT, ALP, GGT, and LDH to normal levels. The effective roles of both IL-35 and THYO presented in this research are consistent with the other findings which reported that administration of *Marrubium vulgare* (Akther et al., 2013), *Muntingia calabura* (Mahmood et al., 2014), and *Auricularia polytricha* (Chellappan et al., 2016) against PAR-induced rats resulted in restoring the liver enzymes to the normal.

Also, due to the fact that a healthy liver is important for the synthesis of certain proteins, hypoproteinemia and hypoalbuminemia are frequently associated with liver damage due to a decrease in protein synthesis as shown in the PAR-treated group. But it restored to normal after treatment with IL-35 or THYO as depicted in Table 1. Toxicity of PAR could lead to excessive heme destruction and obstruction of biliary ducts, triggering hyperbilirubinemia as a result of conjugation reaction inhibition and the escape of unconjugated bilirubin from damaged hepatocyte into the blood (Wolf et al., 1997). After administration of IL-35 or THYO, there was a noticeably decrease of bilirubin level, whereas a higher level of protein was noticed, indicating their protective effects. This could be as a result of the membranes' stability, hepatic parenchyma repair, and hepatocyte regeneration, preventing intracellular enzyme leakage (Vadivu et al., 2008).

According to the current study, the PAR-treated group had considerably higher serum levels of TG, TC, LDL-C, and VLDL-C, but lower serum concentration of HDL-C when compared to the normal control group. However, when PAR was administered in combination with IL-35 or THYO for 3 weeks, levels of TC, TG, LDL-C, and VLDL-C were significantly decreased, whereas HDL-C significantly increased comparing with PAR-induced group. The disruption of lipoprotein metabolism resulting from PAR overdose seems to alter metabolism of cholesterol (Kobashigawa and Kasiske, 1997). The exposure to free acid may decrease hepatic lipoprotein release and enhance esterification of free acids, leading to increased blood levels of TC and TG caused by PAR. When IL-35 or THYO were administered in conjunction with PAR, the hepatoprotective impact was shown as an esterification effect due to the inhibition of free radicals on hepatic cells (Islam et al., 2021).

Enhancement of lipid peroxidation in liver was detected through an elevation of MDA as a result of treatment with high doses of PAR that linked with oxidation of unsaturated fatty acids in the cell membrane (Zoubair et al., 2013). In this experiment, MDA, MYO, NO, and PC levels considerably increased in the PAR-treated rats, while they were decreased to their normal levels in the IL-35- or THYO-treated groups.

Given that SOD, CAT, and GSH play vital roles in safeguarding organisms against the detrimental effects of oxidative stress. They are capable of preventing and scavenge excessive reactive oxygen species accumulation (ROS) (Abduh et al., 2023, Khalaf et al., 2008). Accordingly, impairment of the antioxidant defense mechanism by ROS leads to a decline in SOD, CAT, and GSH, resulting in liver damage (Aladaileh et al., 2019, Khalaf et al., 2008). Our study demonstrated that animal groups given PAR and treated with IL-35 or THYO showed statistical increase in the antioxidant enzymes (SOD, CAT, and GSH). This effect may be directly attributed to the radical-scavenging activity (RSA) exerted by IL-35 and THYO. A recent study revealed a similar increase in antioxidant enzymes (SOD, CAT, and GSH) and decrease in MDA, MYO, and NO after treatment with affective agent as we used in our study (Althunibat et al., 2023, El-Sayed et al., 2021, McGill et al., 2012).

Exploring the outcomes of the present research, it was clear that administration of PAR solely resulted in diminish production of both IgM and IgG antibodies, which have essential role in protecting the organism against infections, but it was significantly reversed. It is well known that elevated oxidative stress, in the absence of proper antioxidant defense, might activate signaling pathways that cause inflammation and cell death, ultimately leading to tissue damage and liver failure (Circu and Aw, 2010, Al-Amarat et al., 2022, Ryter et al., 2007). In fact, the enhanced ROS generation caused by PAR may promote NF- κB activation, which in turn triggers the creation of pro-inflammatory mediators, ultimately causing caspase-dependent apoptotic cell death in the liver (Famurewa et al., 2020, Sun et al., 2021). Treg cells, liver mononuclear cells, Kupffer cells, and inflammatory cytokines (interleukin, TNF-α, and interferon) were produced as a result of certain factors such infections, high-fat eating practices, drug usage, and alcohol (Roderburg et al., 2020). Suppressive cytokines such as TGF-β, IL-10, and IL-35 can directly generate Treg or indirectly stimulate dendritic cells to create Treg (Sojka et al., 2008). Furthermore, According to recent research, IL-35 immunoregulation requires many immune regulatory factors, including TGF-and IL-10, to provide the most potent anti-inflammatory effects (Slawek et al., 2020). This is in agreement with our current research, which shows that the liver's Bcl-2 expression is considerably upregulated, along with increased expression of NF-κB, and caspase-3 and elevated levels of TNF-α, IL-10, and IL-6 levels in PAR-induced animals. After treatment with IL-35 or THYO, significant reductions were detected in anti-inflammatory cytokines, caspase-3, Bcl-2, and cytochrome *C*. Despite the literatures suggests that IL-35 can minimize the lethality of Kupffer cells towards hepatocytes via reducing pro-inflammatory cytokines, such as TNF-α, NO, IL-1β and IL-6 along with enhancing IL-10 anti-inflammatory cytokines, which is not consistent with our findings that revealed reduction in IL-10 after treatment with IL-35 or THYO (Shao et al., 2018).

It has been demonstrated that Bcl-2 works via heterodimerization with pro-apoptotic members of the Bcl-2 family to inhibit the creation of mitochondrial pores, the release of cytochrome *C*, and the activation of apoptosis (Vo and Letai, 2010). The interaction of the cytosolic protein, cytochrome *C*, Apaf-1, d-ATP/ATP, and pro-caspase-9 initiates caspase cascade and leads to the formation of the apoptosome (protein produced during apoptosis) (Hussar, 2022). Research have shown that the creation of ion conduction channels in membranes and local suppression of free radical generation are two possible methods by which Bcl-2 may prevent cell death (MacManus and Linnik, 1997). Bcl-2-associated proteins can either stimulate or block apoptosis, and the interaction between proteins from competing factions determines whether the cell survives or undergoes programmed cell death (Hussar, 2022).

To verify these observations, we explored the molecular mechanisms of the protective effects of IL-35 and THYO's on rats with PAR-induced liver injury. We found that treated groups with IL-35 or THYO upregulated Bcl-2 and IL-10 gene expressions, while downregulated Bax, caspase-3, IL-1β, TNF-α, and TGF-β genes in comparison with PAR-induced group. Prior research investigated the impact of IL-35 on VEGF (vascular endothelial growth factor) and its receptors, Flk-1 and Flt-1, in a collagen-induced arthritic mice model of rheumatoid arthritis revealed that the IL-35 therapy decreased levels of TNF-α and other indicators, including VEGF, Flt-1, and Flk-1 (Wu et al., 2016). Similarly, a previous study employed the drug cyclophosphamide to cause liver damage in mice and then treated them with the flavonoid compound taxifolin had results that were comparable to those of our investigation, which demonstrated a substantial reduction of liver injury biomarkers (NF-κB, TNF-α, IL-10, IL-6, and caspase-3) (Althunibat et al., 2023).

At the same time, histological examination of liver tissues showed the obvious protective effects of both IL-35 and THYO on PAR-induced rats via their ability to restore all tissues histological structure to normal, except mild to moderate hepatocytic hydropic degeneration and minute droplets of fatty globules at centrilobular and mid zonal areas of some hepatic lobules. Intriguingly, the outcome of the present study demonstrated that IL-35 and THYO prevent liver injury through attenuating oxidative stress, hepatic inflammation, fibrosis, apoptosis in tissues, as well as downregulation of Bcl-2, cytochrome *C*, and caspase-3 in parallel with IL-10, IL-6, TNF-α, and NF-κB. In addition, restoring the liver tissues into their normal structure.

## 5 Conclusion

The findings of this study indicate that treatment with that IL-35 or THYO can alleviate PAR-induced liver injury through decreasing the extent of oxidative tissue injury, TC, TG, inflammatory response, hepatocyte apoptosis, and restoring liver tissues and enzymes to the normal levels. These data also suggest that the beneficial effects of IL-35 or THYO are associated with upregulation of Bcl-2 and IL-10 expressions and downregulation of Bax, caspase-3, IL-1β, TNF-α, and TGF-β genes. Further clinical research is therefore necessary to investigate the potential efficacy of IL-35 in preventing and treating PAR hepatotoxicity, as well as other disorders related to inflammation and oxidative stress. To confirm the results of this study, further investigation on the long-term effects of IL-35 or THYO treatment utilizing different dosages and other animal models is strongly recommended.

## Funding

The Deanship of Scientific Research (DSR) at king Abdulaziz University (KAU), Jeddah, Saudi Arabia has funded this project under grant No. (G:614–290-1443).

## Declaration of Competing Interest

The authors declare that they have no known competing financial interests or personal relationships that could have appeared to influence the work reported in this paper.
