# Supplementary material for: Interleukin-35 and Thymoquinone nanoparticle-based intervention for liver protection against paracetamol-induced liver injury in rats
Source: Saudi J Biol Sci. 2023 Sep 6;30(10):103806. doi: 10.1016/j.sjbs.2023.103806 (PMC10519855; doi:10.1016/j.sjbs.2023.103806)
Supplement: Supplementary data 1 [file mmc1.docx]

Supplementary Material

Interleukin-35 and Thymoquinone Nanoparticle-Based Intervention for Liver Protection against Paracetamol-Induced Liver Injury in Rats

Maisa Siddiq Abduh ^a , b^, Sultan Ayesh Mohammed Saghir ^c^, Naif Ahmed Al-Gabri ^d , e^, Ahmad Faheem Ahmeda ^f, g^, Mouaadh Abdelkarim ^h^, Saleh Mohammad Aldaqal ^i^, Mohammed Abdullah Alshawsh ^j , k , ⁎^

# Supplementary Figures and Tables

## Supplementary Figures


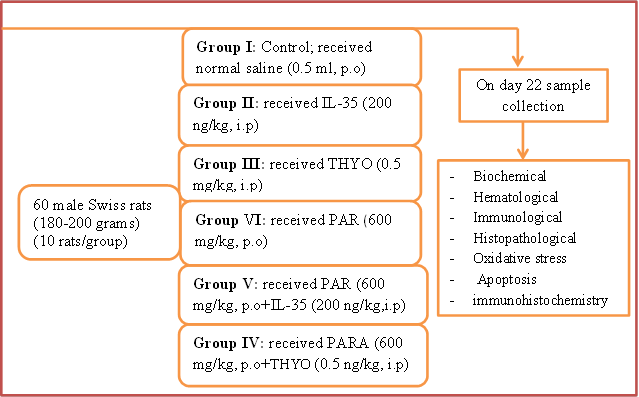


**Supplementary Figure 1.** Scheme of study: Experimental design of the study. IL-35; interleukin 35, (i.p); intraperitoneal, p.o; per oral, PAR; paracetamol, THYO; thymoquinone nanoparticles.

## Supplementary Tables

**Supplementary Table 1.** Primer sequences used for the qPCR analysis

| **Genes** | **Forward (5′-3′)** | **Reverse (5′-3′)** | **Size (bp)** | **Accession No.** |
| --- | --- | --- | --- | --- |
| TNF-α | CCAGAAGCACTAAAGGCGAAGA | CCTTGGCTTTGCTGCTGATC | 82 | AY428948.1 |
| TGF-β | AGGGCTACCATGCCAACTTC | CCACGTAGTAGACGATGGC | 96 | NM_021578.2 |
| Bax | GACTGAGTACCTGAACCGGCATC | CTGAGCAGCGTCTTCAGAGACA | 135 | NM_016993.1 |
| Casps-3 | GAGACAGACAGTGGAACTGACGATG | GGCGCAAAGTGACTGGATGA | 147 | NM_012922.2 |
| IL1β | TGGTGACTCTCCTGGTCTGA | GCACAACTTTATCGGCTTCCA | 86 | XM_005457887.1 |
| IL10 | CTGCTAGATCAGTCCGTCGAA | GCAGAACCGTGTCCAGGTAA | 94bp | XM_003441366.2 |
| Bcl-2 | CGAATTGGCGATGAACTGGA | CAAACATGTCAGCTGCCACAC | 109 | NM_017059.2 |
| GAPDH | TGACGTGGACATCCGCAAAG | CTGGAAGGTGGACAGCGGAGG | 143 | NM_017008.4 |

GAPDH: glyceraldehyde-3-Phosphate Dehydrogenase; Bcl2: B-cell lymphoma 2; Bax: Bcl-2-associated X protein; Casps3: Caspase-3; IL-1β: interleukin-1β; TNF-α: tumor necrosis factor α; IL-10: interleukin-10; TGF-β: Transforming growth factor beta.

**Supplementary Table 2.** IL-35 and THYO attenuates the changes of erythrocytes and leucocytes induced by paracetamol in rat’s model.

| **Test** | **Control** | **IL-35** | **THYO** | **PAR** | **PAR+IL35** | **PAR+THYO** |
| --- | --- | --- | --- | --- | --- | --- |
| **Erythrocytes** | 5.07±0.25 |  |  | 3.38±0.09* | 4.97±0.34^#^ | 4.42±0.28^#^ |
| RBC (1012/L) |  | 4.85±0.07 | 4.97±0.44 |  |  |  |
| HGB ( g/dl ) | 15.07±0.27 | 15.37±0.17 | 15.79±0.44 | 10.17±0.33* | 15.29±0.66^##^ | 14.04±0.65^##^ |
| PCV ( % ) | 41.85±0.48 | 42.96±0.25 | 43.41±0.42 | 34.72±1.27* | 42.41±0.76^#^ | 40.95±1.36^#^ |
| MCV (FL) | 87.05±2.43 | 91.36±1.59 | 89.42±7.35 | 74.96±1.45** | 87.04±3.52^#^ | 91.88±3.14^#^ |
| MCH (pg) | 29.89±0.96 | 32.71±0.79 | 32.44±2.44 | 30.14±1.17 | 30.95±0.89 | 31.9±14.06 |
| MCHC(g/dL) | 35.99±0.30 | 35.79±0.24 | 36.36±0.72 | 36.27±0.36 | 35.99±0.90 | 34.24±0.60 |
| PLT (109/L) | 404.0±29.2 | 463.25±83.7 | 612.5±57.5 | 525.7±36.8 | 498.5±74.3 | 496.75±91.38 |
| **Leucocytes** |  |  |  |  |  |  |
| WBC (109/L) | 8.8±0.8 | 9.09±1.3 | 9.48±1.16 | 5.17±0.6* | 8.7±0.33^#^ | 9.23±1.1^#^ |
| Lymphocytes | 0.84±0.07 | 0.83±0.009 | 0.82±0.06 | 0.83±0.08 | 0.82±0.07 | 0.83±0.08 |
| Eosinophils | 0.01±0.01 | 0.02±0.001 | 0.02±0.01 | 0.01±0.01 | 0.02±0.01 | 0.02±0.01 |
| Neutrophils | 0.08±0.01 | 0.08±0.001 | 0.012±0.01 | 0.08±0.01 | 0.08±0.01 | 0.08±0.01 |
| Monocytes | 0.03±0.01 | 0.04±0.01 | 0.03±0.01 | 0.02±0.01 | 0.03±0.01 | 0.03±0.02 |
| Basophils | 0.02±0.01 | 0.03±0.01 | 0.03±0.01 | 0.02±0.01 | 0.02±0.01 | 0.03±0.01 |

IL-35; interleukin 35, THY-nano; thymoquinone nanoparticles, WBC; white blood cells, RBCs; red blood cells, Hb; hemoglobin, PCV; packed cell volume, MCV; mean corpuscular volume, MCH; mean corpuscular hemoglobin, MCHC; mean corpuscular hemoglobin concentration. Data presented as mean±SEM, One-way ANOVA followed by Tukey's post-hoc tests were used for comparing data at significance level *p*<0.05. **p*˂0.05 and ***p*˂0.01 indicate changes in PAR group compared to the control group, whereas ^#^*p*˂0.05 and ^##^*p*˂0.01 show changes in groups treated with IL-35 or THYO against untreated PAR-induced group.
